# Role of New Hydrophilic Surfaces on Early Success Rate and Implant Stability: 1-Year Post-loading Results of a Multicenter, Split-Mouth, Randomized Controlled Trial

**DOI:** 10.1055/s-0040-1713952

**Published:** 2020-11-26

**Authors:** Marco Tallarico, Nicola Baldini, Fulvio Gatti, Matteo Martinolli, Erta Xhanari, Silvio Mario Meloni, Cervino Gabriele, Lumbau Aurea Immacolata

**Affiliations:** 1 School of Dentistry, University of Sassari, Sassari, Italy; 2 Department of Periodontics and Implantology, University of Siena, Siena, Italy; 3 Department of Biomedical, Surgical and Dental Sciences, Unit of Oral Surgery, San Carlo and San Paolo Hospital, University of Milan, Milan, Italy; 4 Private Practice in Porto Viro, Porto Viro, Italy; 5 Medical Surgical and Experimental Science Department, University of Sassari, Sassari, Italy; 6 Department of BIOMORF, School of Dentistry, University of Messina, Messina, Italy

**Keywords:** dental implants, implant surface, osseointegration, bone quality

## Abstract

**Objective** To compare early implant failure and implant stability of one-stage Hiossen ET III implants with its new hydrophilic (NH) surface, compared with Hiossen ET III implants with the sandblasted and acid-etched (SA) surface at 1-year follow-up.

**Materials and Methods** This study was designed as a split-mouth, multicenter randomized controlled trial aimed to compare SA surface implants (SA group) and NH surface, (NH group). Outcomes were implant and prosthetic survival rates, complications, the insertion torque at implant placement, and implant stability quotient (ISQ) values.

**Results** Twenty-nine patients (mean age 59.9 ± 11.3 years) were treated and followed up to 1 year after loading. No patient dropped out. Fifty-eight implants (29 SA group and 29 NH group) were placed. No implants or prostheses failed and no complications were experienced during follow-up. The mean insertion torque was 40.5 ± 3.23 (38.17–41.83) Ncm in the SA group and 40.48 ± 3.49 (38.02–41.98) Ncm in the NH group ( *p* = 0.981). There was a statistically significant difference at the second week (T2) with higher values in the NH group ( *p* = 0.041). Similar results were found in the maxilla ( *p* = 0.045), but not in the mandible ( *p* = 0.362). A positive correlation was found between initial insertion torque and ISQ with higher value in the NH group (0.73 vs. 0.66).

**Conclusions** NH implants are a viable alternative to SA surface, as they seem to avoid the ISQ drop during the bone remodeling phase.

## Introduction

Modern dentist has an excellent solution to solve patient’s edentulism using dental implants. It has been shown that dental implants have long-term successful outcomes, representing a viable option for clinicians to rehabilitate complete or partial edentulous patients with both fixed and removable solutions. 1 Albrektsson et al introduced the concept of foreign body equilibrium, applied to the osseointegration of titanium dental implants. This equilibrium is an immune-mediated foreign body reaction balance during the biological integration of dental implants into the bone. When this equilibrium moves to a disadvantage periimplant bone loss can occur. 2 Nevertheless, implant failures could still happen in a reduced number of compromised patients, due to the lack of enough understanding of related risk factors.

The causes of bone loss around dental implants and the consequent implant failure may be different and related to implant macro-/microdesign and surface chemical composition, biologic issues, bone quality, surgical technique, host-related factors, and iatrogenic factors. 3 4 5 The failure of a dental implant has been classified as early or late depending on its time of occurrence. 6 Early dental implant failures occur prior to the abutment connection, as consequence of a lack of integration with the bone, 5 6 7 and late failures occurs after prosthetic loading, as consequence of plaque-induced peri-implantitis and/or to occlusal overloading. 8 Osseointegration around titanium implants is a complex biological phenomenon not yet clearly understood. Nevertheless, the surface modifications of titanium dental implants play important roles in the enhancement of osseointegration. With the aim of accelerating and improving the osseointegration process many implant surface treatments were proposed. The surface modification is focused mainly to chemically enhance the roughness of dental implants to increase the appropriate biological response between the living tissues and the dental implants. 9 10 Furthermore, aside of improving osseointegration, these implant surface modifications have been shown to increase cell viability and biocompatibility. 11

This topographical change is achieved by acid treatments, sandblasting, or different mechanisms of oxidization. 8

Sandblasted acid-etched surface (SA) dental implants have a macroroughness achieved with abrasive particles (sandblasting) and micropits obtained by acid etching to improve osseointegration. 8 10 The SA surface provides an appropriate space for osteoblast adhesion, proliferation, and differentiation. 12 This result can be further improved by using a double etching process increasing the surface available for new bone ingrowth, hence greatly improving the mechanical fixation. 13

The more the dental implants used in daily dental practice, the greater the clinical interest becomes in the implants integrating quickly with the bone to be functional. In the last decade, there was a continuous commitment to improve the implant surface to quicken the process of osseointegration and improve its quality. 14 15 Today, the goal is reducing the healing period from 6 to 8 weeks down to 3 to 4 weeks in all the indications. These efforts have been concentrating in improving the bone to implant interface chemically (by incorporating inorganic phases on or into the titanium oxide layer) or physically (by increasing the level of roughness). 16 17

Although shorter healing period was presented in many experimental and clinical studies using sandblasted, large grit, and acid-etched (SLA) surfaces, 18 19 modification of this surface seems to present a stronger bone response than its predecessor. 20 21

The aim of this split-mouth randomized controlled trial was to compare early implant failure and implant stability of one-stage Hiossen ET III implants with its new hydrophilic (NH) surface, compared with Hiossen ET III implants with the well-known SA surface at 1-year follow-up. The null hypothesis was that there is no difference between groups. The null hypothesis was tested against the alternative hypothesis of differences between them. A preliminary report from one center has been published. 10 The following trial was reported according to the CONSORT statement guidelines ( http://www.consort-statement.org/ ).

## Materials and Methods

This study was designed as a split-mouth, randomized controlled trial of parallel groups with two arms and independent outcome assessment when possible, conducted at four centers between November 2017 and May 2018. The protocol was registered in the clinicaltrial.gov (NCT03649100). The 2013 Helsinki declaration was adhered too. The study was performed after approval was received from the Institutional Review Board of the Aldent University, Tirana, Albania (March 2018). All the surgical and prosthetic procedures were performed by one expert clinician at each center.

Any healthy patients, aged 18 years or older, required at least two implants to be rehabilitated with a fixed implant-supported restoration, with a full mouth bleeding and full mouth plaque index ≤25%, with a sufficient bone to allow placement of at least 11.5-mm-long implants, and bone width of at least 6 to 8 mm for the placement of a regular platform Hiossen ET III implant (Deutsche Osstem GmbH, Eschborn, Germany) were included in this study. The exclusion criteria were in Table 1 .

**Table 1:** Exclusion criteria

Positive medical findings (such as stroke, recent cardiac infarction, severe bleeding disorder, uncontrolled diabetes,or cancer).
Psychiatric therapy.
Pregnancy or nursing.
Smoking >10 cigarettes/d.
Insertion torque <30 Ncm.
Untreated periodontitis and/or poor oral hygiene.
Acute and chronic infections of the adjacent tissues or natural dentition.
Previous radiotherapy of the oral and maxillofacial region within the past 5 y.
Postextractive implants (at least 3 mo after tooth extraction).
Absence of teeth in the opposing jaw.
Severe clenching or bruxism.
Severe maxillomandibular skeletal discrepancy.

Patients were informed about the clinical procedures, the materials to be used, the benefits, potential risks and potential complications, as well as any follow-up evaluations required for the clinical study. Patients had to sign the informed consent before including in the study.

A single dose of antibiotic (2 g of amoxicillin and clavulanic acid or clindamycin 600 mg if patients were allergic to penicillin) was administered prophylactically 1 hour before surgery. Patients rinsed with 0.2% chlorhexidine for 1 minute. Local anesthesia will be induced using a 4% articaine solution with epinephrine 1:100,000 (Ubistesin; 3M Italia, Milan, Italy). Implants were placed in the planned anatomic sites using a flapless or a mini-flap approach. Bone density was assessed, according to the Lekholm and Zarb classification, during the drilling phase, based on the clinician’s experience and judgment. Implant site was prepared simultaneously, according to the drilling protocol recommended by the manufacturer (placed at 0.5-mm subcrestal level or deeper according to the bone quality and the soft tissue thickness). The SA surface implants (SA group) or SA surface implants with a newly developed bioabsorbable apatite nanocoating (NH group) were randomized after implant site preparation, immediately before implant placement. Implants used in every group were identical except for the surface treatment. Implants were placed according to a one-stage protocol.

**Fig. 1:**
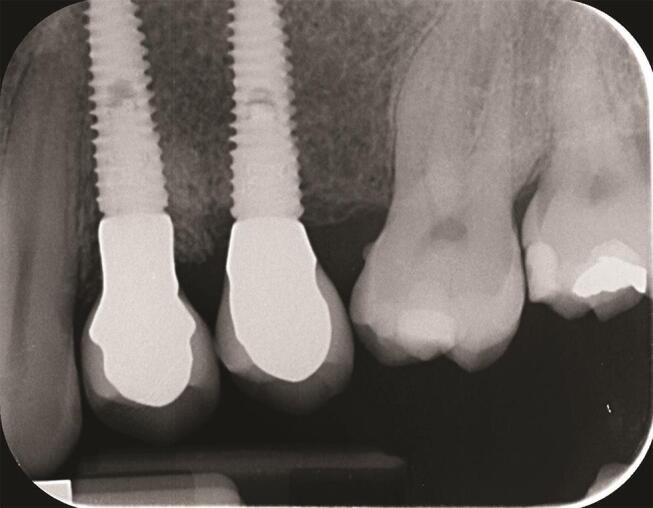
Periapical radiograph at the definitive prosthesis delivery.

Postsurgical analgesic treatment was performed with ibuprofen 600 mg, which was administered twice a day for 2 days after the surgery, and later on, if required. Periapical radiographs were taken with a customized holder at implant placement, at the definitive prosthesis delivery ( Figs. 1 2 ), and then yearly ( Figs. 3 4 ). Two to three months after implants placement patients receive single screw-retained restorations.

**Fig. 2:**
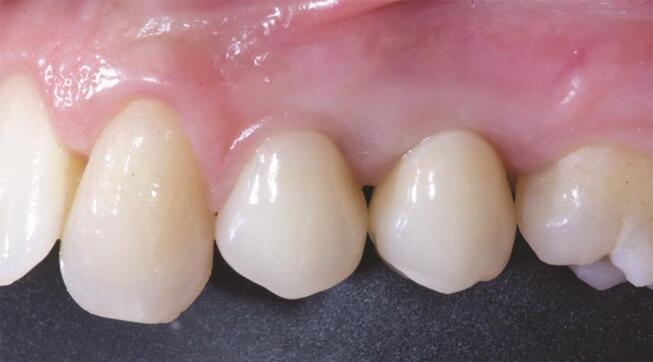
Clinical picture at the definitive prosthesis delivery.

**Fig. 3:**
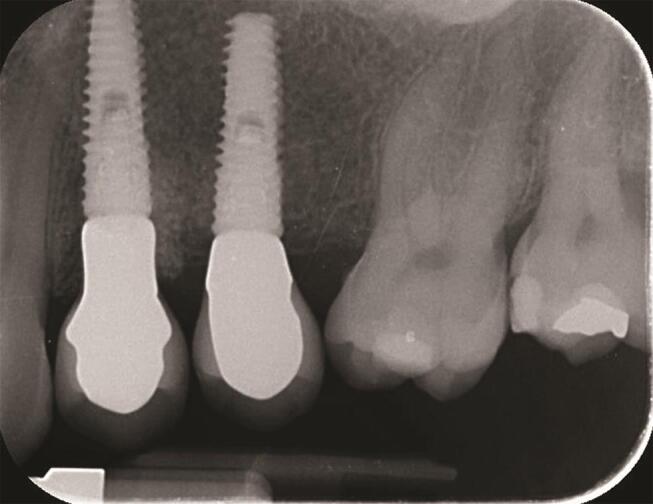
Periapical radiograph at the 1-year follow-up.

**Fig. 4:**
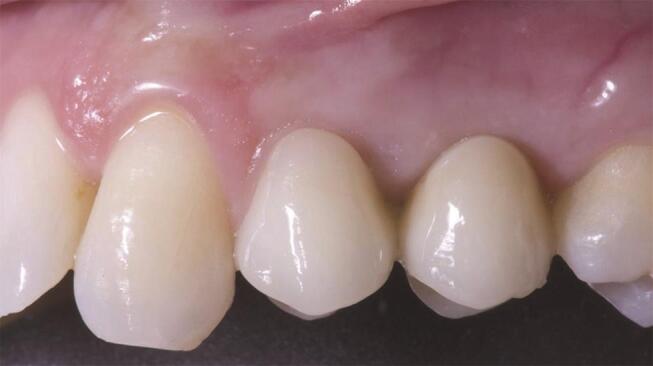
Clinical picture at the 1-year follow-up. ISQ, implant stability quotient.

The outcome measures were implant and prosthetic survival rates, any biological or mechanical complications at implants that occurred during the entire observation period, the insertion torque at implant placement, and the implant stability quotient (ISQ).

Success rates of the implants and prostheses were evaluated by an independent assessor (E.X.). An implant was considered a failure if it presented mobility, assessed after the osseointegration period by tapping or rocking the implant head with the metallic handles of two instruments, progressive marginal bone loss or infection, or any mechanical complications rendering the implant unusable, although still mechanically stable in the bone. A prosthesis was considered a failure if it needed to be replaced with another prosthesis.

Biological (pain, swelling, suppuration, etc.) and/or mechanical (screw loosening, fracture of the framework, the veneering material, etc.) complications occurred during the follow-up period. Complications were evaluated and treated by the same surgeon (M.T.).

Insertion torque was recorded at implant placement by the same surgeon (M.T.) using the iChiropro surgical unit (Bien-Air, Bienne, Switzerland).

The insertion torque values of the implants were measured and recorded at implant placement, using the same surgical unit used to place the implants.

The ISQ was measured and recorded using a smart peg (Type 47 cod. 100478, Osstell, Gothenburg, Sweden) connected to the implants, and the Osstell Mentor device (Osstell). Measurements were taken at implant placement, and every week up to 8 weeks after implant placement. In case of ISQ value <55 or in case of implant mobility, healing abutment was replaced with a cover screw and the implant was left to heal submerged for at least 6 weeks.

A blind outcome assessor collected the data (E.X.), according to a previously published study. 3

A pregenerated random list, consisting of a randomized sequence of consecutive numbers matching the two different procedures within group A or group B, was created using random number generator pro 1.91 for Windows (Segobit Software; www.segobit.com ). Opaque envelopes containing the randomization codes were sequentially numbered and sealed. According to a pre-generated list, an independent consultant, not previously involved in the trial, prepared all the envelopes and then opened immediately after implant sites preparation. Site one was defined the site with the lower sextant number and the most mesial. Patients and statistician were blinded, while doctor not due to the different opacity of the implant surface. Patient data were collected in an Excel spreadsheet (Microsoft) that reflected the parameters in the patient records. The data were exported into SPSS software for Mac OS X (version 22.0; SPSS, Chicago, Illinois, United States), for the statistical analysis. Descriptive analysis was performed for numeric parameters using means and standard deviations (95% confidence interval). Complications and failures were compared using the Fisher’s exact test. Comparisons between groups (SA vs. NH), and between jaws (maxilla vs. mandible) were made by unpaired *t* -test, while the comparison between baseline (T0) and the last follow-up (T8) was made by paired *t* -tests to detect any change during the follow-up. Pearson’s correlation coefficient was used to evaluate the correlation between insertion torque at implant placement and ISQ value 8 weeks after implant placement. All statistical comparisons were two-tailed and conducted at the 0.05 level of significance. The patient was used as the statistical unit of analysis.

## Results

Only three out of four centers managed to recruit and treat patients according to the study protocol. Initially, 39 patients were screened but six patients were not included because they did not have sufficient bone to allow placement of 11.5-mm-long and 4-mm diameter implants; two patients were not included because they did not want to participate in the study; and the other two patients were heavy smokers. A total of 29 patients (22 females and seven males, with a mean age at implant insertion of 59.9 ± 11.3 years) were treated according to the allocated interventions and followed up to 1 year after loading. No patient dropped out. A total of 58 implants (29 with SA surface and 29 with SA surface with the newly developed bioabsorbable apatite nanocoating) were placed. Eighteen patients were rehabilitated in the maxilla and 11 in the mandible. One-year after loading, no implant and no prosthesis failed. Two weeks after implant placement, two Hiossen ET III SA implants showed a small mobility with an ISQ values lower than 55 (49 and 51, respectively). The healing abutments were replaced with cover screws and the implants were left to heal undisturbed up to 8 weeks after their placement. Nevertheless, no statistically significant difference was reached ( *p* = 0.491). In both the implants, the healing abutments were replaced with a cover screw and the implants were left to heal submerged for 6 weeks (up to 8 weeks after implant placement).

The mean insertion torque ranged between 35.0 and 45.0 Ncm (mean of 40.5 ± 3.23 [38.17–41.83] Ncm in the SA group and 40.48 ± 3.49 [38.02–41.98] Ncm in the NH group). The difference between groups was not statistically significant ( *p* = 0.981).

**Fig. 5:**
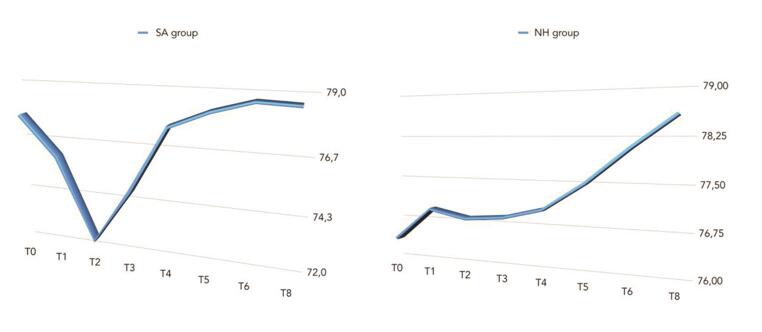
Overall comparison of mean ISQ values between groups. ISQ, implant stability quotient.

The comparison between ISQ values was reported in Table 2 Figs. 5 6 7 .

**Table 2:** The ISQ values between and within groups

	T0 ( *n* = 29)	T1 ( *n* = 29)	T2 ( *n* = 29)	T3 ( *n* = 27)	T4 ( *n* = 27)	T5 ( *n* = 27)	T6 ( *n* = 27)	T8 ( *n* = 29)	Difference T8–T0	*p* -Value
SA	77.8 ± 5.7 (75.8–82.2)	76.1 ± 6.0 (74.1–80.9)	72.7 ± 9.0 (68.9–79.1)	75.0 ± 7.0 (71.0–79.0)	77.7 ± 4.9 (75.3–80.7)	78.3 ± 3.6 (75.9–80.1)	78.8 ± 3.7 (76.1–80.4)	78.7 ± 4.3 (76.8–81.7)	1.0 ± 4.4 (2.0 to 3.0)	0.266
NH	76.4 ± 5.7 (71.8–78.2)	77.0 ± 5.5 (71.9–78.1)	76.9 ± 4.9 (72.2–77.8)	76.9 ± 4.9 (75.2–80.8)	77.1 ± 4.7 (75.3–80.7)	77.5 ± 4.2 (75.6–80.4)	78.1 ± 4.3 (75.6–80.4)	78.6 ± 3.8 (77.8–82.2)	1.9 ± 3.9 (−1.2 to 3.2)	0.019 ^a^
Difference	1.3 ± 6.5 (−1.7 to 5.7)	0.8 ± 5.1 (−1.0 to 2.9)	4.2 ± 9.1 (−7.2 to 3.2)	1.9 ± 4.4 (−4.0 to 1.0)	0.5 ± 4.1 (−3.3 to 1.3)	0.7 ± 3.1 (−1.7 to 1.7)	0.5 ± 2.7 (−1.5 to 1.5)	0.1 ± 3.9 (−2.5 to 2.0)	–	–
*p* -Value	0.393	0.597	0.041 ^a^	0.258	0.662	0.473	0.550	0.919	–	–
Abbreviations: ISQ, implant stability quotient; NH, new hydrophilic; SA, sandblasted and acid etched. ^a^ Statistically significant.										

**Fig. 6:**
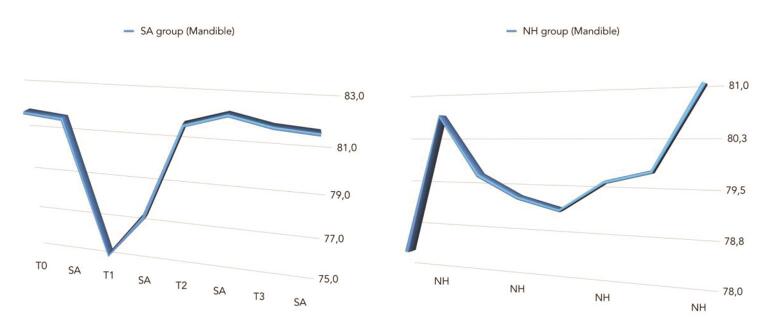
Comparison of mean ISQ values between groups (in the mandible). ISQ, implant stability quotient.

**Fig. 7:**
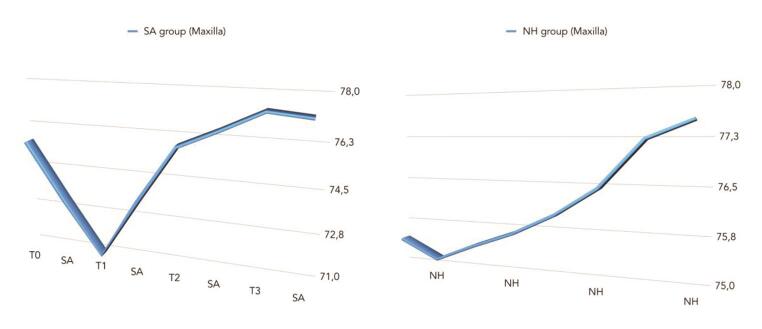
Comparison of mean ISQ values between groups (in the maxilla). ISQ, implant stability quotient.

There was a statistically significant difference between groups at the second week after implant placement (T2) with higher values in the NH group ( *p* = 0.041). Similar results were found in the maxilla ( *p* = 0.045), but not in the mandible ( *p* = 0.362). Overall, the ISQ values improved in both groups during the entire follow-up (8 weeks), with statistically significant difference in the NH group ( *p* = 0.019), but not in the SA group ( *p* = 0.266). A positive correlation was found between initial insertion torque and ISQ with higher value in the NH group (0.73 vs. 0.66). Correlation was stronger in the mandible (SA = 0.71; NH = 0.86) compared with the maxilla (SA = 0.52; NH = 0.55).

## Discussion

Nowadays there is a strong effort in improving the bone to implant interface modifying dental implant surface to improve bone integration and reduce the timing of this process to help clinician in the treatment of edentulous patients.

Exactly in this context our study was oriented, in fact this split-mouth randomized controlled trial was aimed to compare early implant failure and implant stability of one-stage Hiossen ET III implants with its NH surface, compared with Hiossen ET III implants with the well-known SA surface up to 1-year of follow-up. The null hypothesis of no difference was partially rejected in favor of the alternative hypothesis of difference between groups.

Overall, the mean ISQ improved in both groups during the 8 weeks of follow-up, but the values were with statistically significant difference only in the NH group ( *p* = 0.019). The reason was partial due to the fact that 2 weeks after implants placement the mean ISQ value was statistically significant higher in the NH group compared with the SA group ( *p* = 0.041). A possible explanation could be the unexpected values of ISQ in the SA implant group caused by two implants that showed values <55 with slightly implant mobility. As a consequence, implants were left to heal submerged for the next 6 weeks. Nevertheless, it can therefore be assumed that implants with the hydrophilic surface (NH) seem to reduce complications avoiding the ISQ drop back during the remodeling phase allowing accordingly benefits in immediate loading, poor bone quality, post-extractive and maxilla. The main limitations of the present randomized controlled trial are the small sample size and the short-term follow-up. Unfortunately, one center did not participate to the study, contributing to the small sample size.

The ongoing effort of dental companies to improve the interface between bone and implant surface to speed up the process of osseointegration has been proposed by researcher and dental implant companies, and data underlined in this paper, especially for the NH surface, confirm the chance to reduce time in implant therapy. Today, primary implant stability and absence of micromovements still remain two of the main prerequisites for obtaining a stable osseointegration and the achievement of long-term high-success rates. 22 23 On the contrary, if during the first healing period of the implant the primary stability is insufficient, early implant failure can occur. 24 25

In the present study, similar statistically significance was found in the maxilla ( *p* = 0.045), but not in the mandible ( *p* = 0.362). To minimize the change of early implant failure, during the last decades it has been suggested that implants should be kept load-free during a healing period of 3 to 4 months in mandibles and 6 to 8 months in maxillae. 26 Nowadays, the more the implants are used in clinical routine, the greater the clinical interest becomes in the implants integrating quickly with the bone to be functional. An ongoing effort to improve the interface between bone and implant surface to speed up the process of osseointegration has been proposed by researcher and dental implant companies, modifying implant surface roughness and topography. 26

To better understand dental implant roughness it is commonly divided, depending on the dimension of the measured surface features, into macro-, micro-, and nano roughness. All these kinds of roughness and topography have direct consequences on bone response during the healing period of tissues around dental implants. 27 28 29 Nowadays, it is well known that the implant roughness improves osseointegration and the majority of implant types are sandblasted and/ or acid-etched to increase their surface texture. 24 Furthermore the nanometer roughness has the main role in the adsorption of proteins, adhesion of osteoblastic cells, and thus the rate of osseointegration. 30 Furthermore, Schwarz et al showed that hydrophilic surfaces enhance the angiogenesis process when early stages of osseointegration occur. 31 32 33 Actually, fast vascularization seems beneficial for bone formation because osteogenic cells have been observed to arise from pericytes adjacent to small blood vessels. 23 34 In a review of Wennerberg et al, a little clinical evidence was found to clearly state a preference for SLActive over SLA implant. 35 36 At 1-year follow-up, there was a high survival rate (100% for SLActive vs. 96% for SLA implants) and low crestal bone loss <0.4 mm in both groups with no significant difference.

Recent literature suggest that an optimal insertion torque could be around 30 Ncm to obtain a successful and durable osseointegration, which is also sufficient to allow both conventional and immediate occlusal loading of dental implants. 37 The reason why dental implant companies and researchers are focused on implant design improvements and surface modification is to help both clinicians and patients to fasten implant surgery and prosthetic timing, 26 38 39 40 41 42 43 44 for same reasons, researchers are focused on digital dentistry and in developing guidelines in implant dentistry. 45 46 47

In the present study, a positive correlation was found between initial insertion torque and ISQ with higher value in the NH group (0.73 vs. 0.66). Correlation was stronger in the mandible (SA = 0.71; NH = 0.86) compared with the maxilla (SA = 0.52; NH = 0.55). The clinical implication from this randomized controlled trial may be that implants with NH surface modification could be an important option when treating patients, especially when the timing of loading is crucial. Considering the positive correlation found between high torque insertion and an increased ISQ level, and better results in the maxilla, this surface treatment could be a viable treatment option in case of immediate loading, poor bone quality (such us posterior maxilla), postextractive implants, or high risks patients, such us immunocompromised patients or heavy smokers.

## Conclusions

Considering the limitation of this multicenter, split-mouth, randomized controlled trial, NH implants are a viable alternative to SA surface, as they seem to avoid the ISQ drop during the remodeling phase. It can be beneficial in immediate loading, poor bone quality, postextractive implants, smoking, and immunosuppression. Further studies are needed to improve the number of patients and long-term follow-up.
